# Geometry Design, Principles and Assembly of Micromotors

**DOI:** 10.3390/mi9020075

**Published:** 2018-02-11

**Authors:** Huanpo Ning, Yan Zhang, Hong Zhu, Andreas Ingham, Gaoshan Huang, Yongfeng Mei, Alexander A. Solovev

**Affiliations:** 1 Department of Materials Science, Fudan University, 220 Handan Road, 200433 Shanghai, China; h.ning@dhu.edu.cn (H.N.); zhangyan19861103@163.com (Y.Z.); 17110300005@fudan.edu.cn (H.Z.); yongfeng.mei@gmail.com (Y.M.); 2 Department of Biology, University of Copenhagen, 5 Ole Maaløes Vej, DK-2200, 1165 København, Denmark; andreas.ingham@cpr.ku.dk

**Keywords:** micromotor, microengine, micropump, catalyst, complex, self-assembly, fluidic, collective, sub-system, chemoton

## Abstract

Discovery of bio-inspired, self-propelled and externally-powered nano-/micro-motors, rotors and engines (micromachines) is considered a potentially revolutionary paradigm in nanoscience. Nature knows how to combine different elements together in a fluidic state for intelligent design of nano-/micro-machines, which operate by pumping, stirring, and diffusion of their internal components. Taking inspirations from nature, scientists endeavor to develop the best materials, geometries, and conditions for self-propelled motion, and to better understand their mechanisms of motion and interactions. Today, microfluidic technology offers considerable advantages for the next generation of biomimetic particles, droplets and capsules. This review summarizes recent achievements in the field of nano-/micromotors, and methods of their external control and collective behaviors, which may stimulate new ideas for a broad range of applications.

## 1. Introduction

### 1.1. Mechanical Machines: Simple, Complex, Chaotic, and Fluidic

Beginning with illustrative examples in mechanical engineering of simple, complex, predictable, chaotic machines and extending to miniature systems on the scale of single atoms, this review and the cited references contained within stand alone as a comprehensive introduction to nano-/micro-motors (NMs), intended to answer questions about possible future design directions and considerations required for the next generation of nano-/micro-motors. This review discusses (i) new NMs’ geometries, surfaces, fabrication, fuels, efficiency, applications; (ii) external control of NMs using magnetic, acoustic, electrical fields, light for cargo delivery and on-chip integration; (iii) swarming, collective, adaptive behaviors using chemically and external field triggered NMs; and (iv) bio-soft-hybrid NMs for roving sensors, drug delivery applications. We also propose a new system by integrating sub-systems for an assembly of “synthetic cells” using microfluidics. Our aim is to spark new collaborations across disciplines and open new exciting horizons in the field of nano-/micro-machines, which is currently in its infancy, yet in high demand.

The genesis of the man-made NMs (nano-/micro-engines, motors rotors, pumps) research field began about 15 years ago [1,2,3,4,5,6,7]. Inspired by the shape and geometry of biological motors like proteins, flagellum, and related cellular motility, small machines are also generated to duplicate the capabilities of large-scale motors, engines and rotors. The tremendous promise of NMs is to have a profound influence on the environment, biomedicine, clean energy, cleaning of the environment, and on-chip integration. Analogous to the manufacture of an automobile in an assembly line process of the automotive industry, which began around 300 years ago, small machines require the integration of many innovations, such as high efficiency motors, geometrical optimization, reduced time of fabrication, improved methods of fabrication, and precision assembly of components in the assembly line.

**Figure 1:**
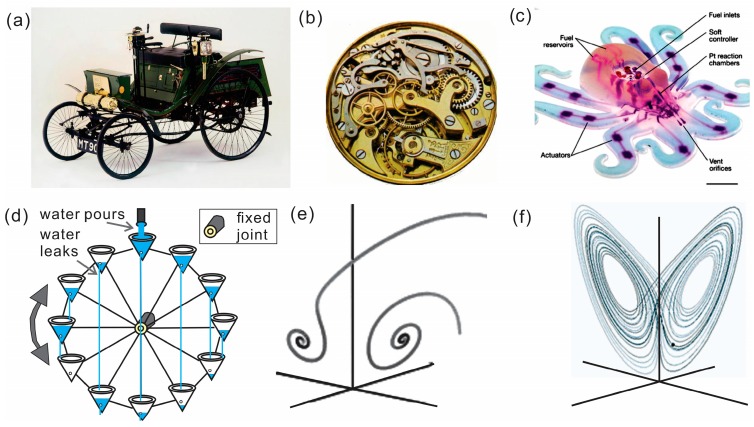
Mechanical, soft, complex (predictable) and complex (aperiodic, chaotic) macroscale machines. (**a**) one of the first modern car models (credit: Arnold Benz Motor Carriage, 1896). Reproduced from [12]; (**b**) a clockwork mechanism consisted of multiple mechanical elements with well understood forces, interactions and dynamics between mechanical components using reductionism approach. Reproduced from [13]; (**c**) biomimetic octobot that includes fluidic logic gates, powered by decomposition of chemical fuel. Reproduced with permission from [8], copyright (2016) Springer Nature; (**d**) chaotic Lorenz water wheel, which represents a classical example of the long-term unpredictability of a deterministic nonlinear dynamic system. Here, water pours into the buckets at a steady rate and gives the wheel energy, while gravity and water leakage out of each bucket removes energy from the system; (**e**) phase portrait of a typical system that reaches its equilibrium state. Reproduced from [14]; (**f**) a constant pouring of water leads to aperiodic oscillations of the wheel forth and back and an appearance of aperiodic/chaotic motion according to the Lorenz system. Reproduced from [15].

Moreover, designed micro-/nano- motors, rotors, engines and pumps represent a microscopic analogy of large-scale man-made machines [1,2,3,4,5,6,7]. Classical mechanics provides very accurate results, when describing the motion of macroscopic bodies, including joint machine parts, which are not extremely heavy nor very small with much lower speeds than the speed of light. According to the definition in classical mechanics, a simple machine consists of only one part and a complex machine consists of at least two or more machine parts working together. For instance, a car, or a clockwork mechanism, (Figure 1a,b) are classical examples of complex machines. Another illustrative example of a complex machine is the recently fabricated octobot, as shown in Figure 1d. This soft robot mimics movements of an octopus with embedded integrated fluidic logic circuits, which are powered by chemical decomposition of hydrogen peroxide into oxygen and water [8]. 

We currently live in an era of reductionism that provides many new opportunities in developing complex machines with deterministic behavior. In reductionism, a complex machine’s parts are broken down into smaller components and, with these simple individual parts, forces and interactions can be understood using classical equations of motion, thus the motion of the composite complex system can be understood. It is a revolutionary mode for understanding complex systems, and thus very useful for having complex machines with easily predictable functions. However, it is already known that biological systems cannot be explained using this reductionist approach. According to Sapolsky [9], bio-systems are non-additive and nonlinear bifurcating systems. Additionally, there are not a sufficient number of individual components to explain their collective function (e.g., not enough neurons to recognize faces; not enough genes to program capillaries in a body). Moreover, the system itself can be a variability rather than the accompanied noise, which often needs to be avoided for obtaining reliable data. Thus, while knowing the initial state and conditions, there exists no predictability about determining the final state [9]. Luckily, the discovery of chaotic systems by Lorenz in 1962 led to a better understanding of new dynamic systems for preparing weather forecasts, understanding the combined motion of double pendulum and knowing the chaotic rotation of a water wheel (Figure 1d), all of which are examples of unpredictability of nonlinear dynamic systems [10]. It shows remarkable examples that not every dynamic state is linear and predictable as was previously accepted before the discovery of chaos. To illustrate the chaotic nature of nonlinear dynamical systems, let’s consider the chaotic Lorenz wheel, which is driven by both gravity and the pouring of water into buckets as shown in Figure 1d. As water pours into the buckets at a steady rate, the wheel rotates. Complicating matters, each bucket has a small hole at its bottom. As water leaks from the bottom of each bucket, energy is removed from the system, making the system oscillate. However, if only a small amount of water/energy is added, the wheel rotates and stops after some time, leading to a system’s static state (Figure 1e). When larger amounts of water/energy is added, the wheel starts to oscillate forth and back, leading to an appearance of unpredictable dynamic states (Figure 1f). In other words, although the entire structure of the machine can be understood, it is not possible to predict the dynamic behavior from its initial conditions, linear interactions and energy flow. According to Sapolsky, there is no real “static answer” in chaotic systems, noise does not depend on the quality of our reductive tools or if we observe the system closer—variability is the phenomenon at any scale. In other words, chaotic machines can escape from the control of an operator which is, obviously, not desirable for utility. Biological systems are examples of dynamic systems, which operate on different and far more complex principles than a mechanical clock. It remains a speculative question, if principles of more complex NMs can be based on purely predictable classical mechanics or we should search for spontaneous behaviors with so called “emergent properties”, nonlinear interactions and self-organization in hierarchical structures with feedback loops between different levels [11].

### 1.2. Small Machines from Atoms to Nano-, Micro-, and Meso-Particles

**Figure 2:**
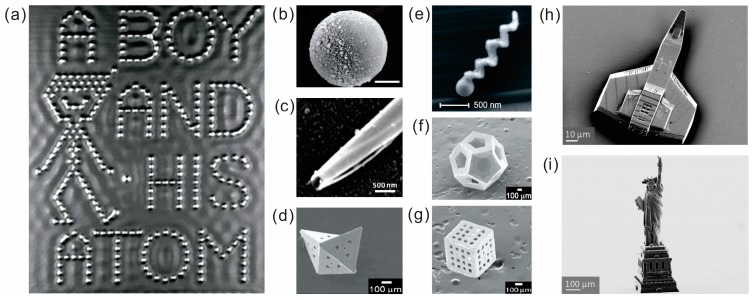
Scanning Tunneling Microscopy and Scanning Electron Microscopy images of fabricated small machines ranging from atoms to nano-, micro- and meso-particles. (**a**) the smallest movie created by International Business Machines Corporation (IBM), entitled “A boy and his atoms”, it is based on nanomanipulation of atoms using scanning tunneling microscopy in ultra-high vacuum and low temperature conditions. Reproduced with permission from [16]. Reprint Courtesy of International Business Machines Corporation (Armonk, NY, USA) © 2013 International Business Machines Corporation. (**b**–**g**) three-dimensional micro-/nanoarchitectures with different shapes: (**b**) spherical Janus microparticle (reproduced from [20]). This work is licensed under the Creative Commons Attribution 4.0 International License [21]. (**c**) a microtube based on rolled-up nanomembrane (image courtesy of D. Gracias); (**d**) self-assembled irregular octahedron using surface tension (Reproduced with permission from [22], copyright (2010) Jon Wiley and Sons); (**e**) individual screw with nanostructured helicity, prepared by glancing angle deposition method (Reproduced with permission from [23], copyright (2009) American Chemical Society); (**f**) self-assembled dodecahedron (Reproduced with permission from [22], copyright (2010) Jon Wiley and Sons); (**g**) fabricated cubic container with holes (Reproduced with permission from [22], copyright (2010) Jon Wiley and Sons); (**h**,**i**) fabrication of complex shapes, micro-airplane and micro-statue of Liberty, enabled by advanced laser photolithography. Images are courtesy of Nanoscribe GmbH (Eggenstein-Leopoldshafen Germany).

Technology has already advanced in both top-down and bottom-up fabrication methods. Atoms are the smallest stable building blocks and are known constituents for construction of machines. According to classifications based on size, nanoscale (<100 nm), microscale (100 nm–100 μm) and mesoscale (>100 μm) particles contain increasingly larger number of atoms, respectively. Small machines with atomic or molecular size can cross the border between classical and quantum mechanical regimes and include many biological cases. Figure 2a shows an image from a movie created by International Business Machines Corporation (IBM), entitled “a boy and his atoms”, where a scanning tunneling microscopy was used to position individual carbon monoxide (CO) atoms on the surface of copper at low temperature, creating the smallest known robot [16]. When well-controlled conditions such as temperature, vibrations and pressure are achieved, atoms can be moved, assembled on the surface causing ripples or disturbances in the electronic density. Although IBM’s “nanobot” cannot exist (yet) in ambient conditions, it demonstrates inspiring capability of new technology based on the smallest building blocks. Our bodies consist of atoms, ions and molecules and perhaps, nano- and Angstrom-scale “motors” made of individual atoms, ions and molecules are the most interesting to consider, not only because of their huge applications, but because of their unique properties, where fundamental laws of quantum mechanics dominate. For instance, wave-particle duality, uncertainty principle, quantization of energy levels, molecules and emission–absorption, i.e., “communication” using photons, and phonons become apparent. For instance, according to Heisenberg’s uncertainty principle, we are not able to measure simultaneously, with high precision, a particle’s position and momentum. This inability to know a particle’s coordinates and momentum simultaneously remains a problem based on the wave-like nature of a particle and not a problem with today’s measurement instruments, neither it is a reflection of the equality of experimental methods. Today, man-made nano-/micromachines are relatively large (10 nm–100 µm) in comparison to atomic structures. The largest known atom (cesium) has the diameter 0.53 nm that is approximately 20 times smaller than the diameter of smallest nanomotor, reported so far. When accepting matter as a continuum approximation, scaling laws can provide useful calculations for engineering of micromachines [17]. However, for truly nanoscale, as well as for molecular and atomic dimensions, scaling laws can be misleading due to an appearance of quantum size-confinement effects, where the mean free path, uncertainty principle, quantization of energy and radiation become significant. Remarkably, many tunable nanomaterials properties can be integrated into small machines/motors, tailored by changing both the dimension of NMs’ layers, segments and their chemical compositions, leading to tunable reactivity, optical, magnetic, electronic, thermodynamic and mechanical properties [18,19].

Early research in the field of NMs began with NMs having characteristic dimensions that were several orders of magnitude larger than atoms—nano-/microparticles (Figure 2b–g). Self-electrophoretic bimetallic nanomotors [2,3], tubular micromotors made of rolled-up nanomembranes were fabricated using different combinations of inorganic catalytic/non-catalytic materials [5,6]. Gracias and co-workers demonstrated more complex 3D shapes using self-folding 2D layers into 3D architectures [22]. Recently, a new two-photon laser lithography enabled fabrication of almost any complex shapes/geometries of objects at the microscale (Figure 2h,i). For example, Figure 2h,i shows the smallest space ship and the Statue of Liberty. In the next step, these complex geometries can be coated with catalytic layers and placed in a fuel solution to achieve self-propelled motion. However, although complex shapes are achievable, it is unlikely that more complex NMs consisted of many mechanical moving parts will be utilized in the near future due to well-known problems of high viscosity of fluids at low Reynolds number, high surface tension and stiction between different parts due to short range forces (e.g., hydrophobic, electrostatic, Casimir).

The development of micro-/nano- motors, rotors, engines and pumps was inspired by biological motor proteins, jet propelled bacteria, flagella, cilia, shape changing and other impressive motile principles used in biology. In living systems, real chemical nanomachines are large macromolecules (enzymes), which catalyze many processes in organisms. Miniaturization in the design of man-made machines lead to multiple advantages, including (1) low weight; (2) fast performance; (3) less inertia, less mass; (4) less energy required to function; (5) increased strength to weight ratio; (6) increased power density, small power consumption and high energy conversion efficiency (chemo-mechanical coupling); (7) precise control of movement at the nano-/microscale; and (8) integration of a large number of devices in a small volume of space, which can enable breakthrough applications. Versatile nanodevices are not perturbed by thermal vibrations due to their high resonant frequencies (the resonant vibration is inversely proportional to the object mass) [23]. For instance, while enzymes can accomplish millions of operations per second, which is not possible by macroscopic robots. According to scaling laws, small machines are relatively stronger than larger machines (strength/weight = 1/D, where D is a characteristic dimension), such as S/W ratio of individual micromotor can be up to 10^6^ times higher in comparison to a macroscale motor) [24].

Bio-nanomotors have been considered promising prototypes for constructing of man-made chemically-actuated micro-/nano- motors, engines, rotors, pumps, powered by catalytic reactions and controlled using external fields [25,26,27,28,29,30,31]. NMs with different aspect ratios, shapes, materials, and fuels can effectively overcome Brownian diffusion and high viscosity of fluids at very low Reynolds number [32,33]. Swarming and collective “chemo- and phototactic” motion of nano-/microparticles were observed in chemical fuels and external fields. Gracias and co-workers noted, that in comparison to already well-established field of Micro-Electro-Mechanical-Systems (MEMS), the main difference being that the Micro-Chemo-Mechanical-Systems (MCMS) are triggered directly by chemistry, similar to biological motor proteins, as opposed to electro-mechanical energy conversion typically used to actuate conventional MEMS [34]. Nano- and micromachines have bright potential in analytical chemistry, sensing, migration, capture, delivery, and separation [35]. Today, several groups demonstrated design of the next generation of NMs at the smallest scales. Fischer and co-workers demonstrated helical nanopropellers [36], and Mei’s group set the next world record for the smallest jet engine consisting of TiO_2_/Pt nanotubes with the diameter as small as 30 nm [37]. External methods to power and control motion of NMs using versatile fuel-free magnetic and electric fields can be used [38]. It is important to mention that catalytic and biocatalytic reactions can enable applications of simpler and more environmentally friendly fuels than used today in gasoline engines, fuels cells or batteries. The fundamental mechanism of energy transduction can shed light on discovery of clean energy nanogenerators.

In the final part of our review, we concentrate on future generations of NMs: bio-hybrid-NMs for biosensing, drug delivery and, importantly, their fabrication and assembly using microfluidic technology, inspired by fundamental concepts concerning microfluidic machines proposed by Ganti [39], who stated two important concepts: (1) human technology is still unable to manipulate energy pathways using chemical means, rather than mechanical or electrical means. This represents a major difference between biological living and synthetic man-made machines; and (2) fluidic space of biological cells is free of mechanical constraints. Next, microfluidic techniques can provide tremendous flexibility in designing soft and fluidic systems. For instance, operation of crawling cells is not disturbed by stirring and diffusion of their internal components. While approaching functionalities inherent to biosystems, an exceptional opportunity exists to realize integration of multifunctional sub-systems using established microfluidic techniques to explore, optimize and mass-produce customized nano-/micromotors or “synthetic cells” with desired motive power and specific utility in very short time.

## 2. New Materials, Geometries and Fuels for Autonomous Motion

**Figure 3:**
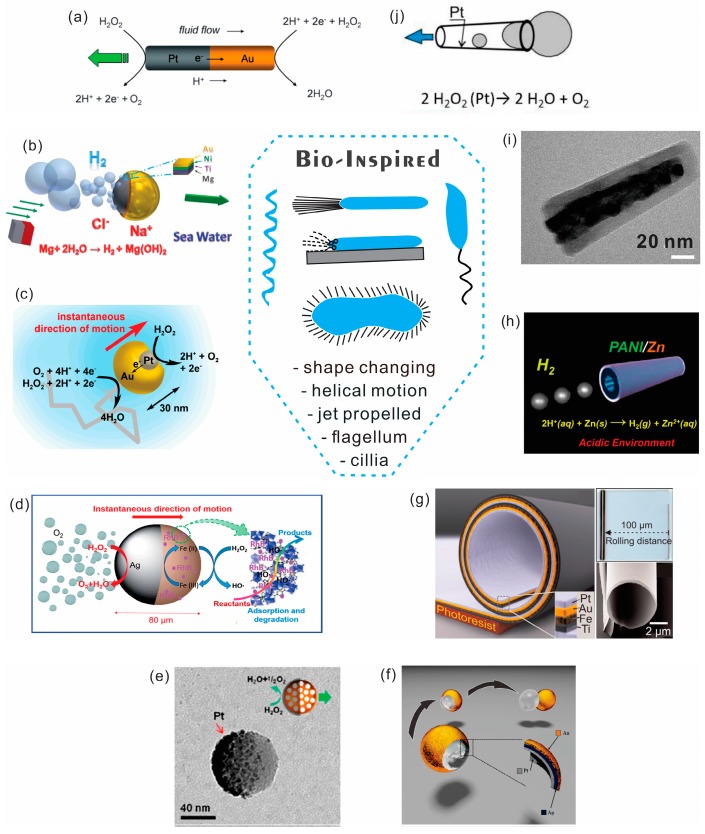
Several illustrative examples are shown: self-propelled nano-/micro-motors (NMs) with different materials, size, geometry, fuels and mechanisms of propulsion. (**a**) state-of-the-art bimetallic nanowire-based nanomotor driven by self-electrophoresis. Reproduced with permission from [40], copyright (2009) Royal Society of Chemistry; (**b**) hydrogen bubbles propelled magnesium spherical micromotor. Reproduced with permission from [41], copyright (2013) Royal Society of Chemistry; (**c**) one of the smallest reported Janus NM with diameter as small as 30 nm. Reproduced with permission from [42], copyright (2014) American Chemical Society; (**d**) metal-organic framework (MOF) based NM specifically designed for water purification [43]; (**e**) one of the smallest mesoporous NM with diameter around 80 nm. Reproduced with permission from [44], copyright (2015) American Chemical Society; (**f**) nanoshell-based NM with microcavity ideally suited for nucleation and generation of bubbles. Reproduced with permission from [45], copyright (2013) American Chemical Society; (**g**) the first example of fabricated tubular NM, made of rolled-up inorganic/catalytic nanomembranes. Reproduced with permission from [7], copyright (2009) Jon Wiley and Sons; (**h**) hydrogen bubbles driven NM in acidic environment. Reproduced with permission from [46], copyright (2012) American Chemical Society; (**i**) the smallest nanojet engine reported to date with diameter as small as 30 nm. Reproduced with permission from [37], copyright (2017) Jon Wiley and Sons; (**j**) schematic image of catalytic tubular microcavity/tube that is ideal for nucleation, growth and recoil of microbubbles, leading to effective bubble-induced pumping mechanism and ultra-high speeds of NMs.

Autonomous motion of NMs was initially inspired by motor proteins and self-propelled motile cells using cilia, flagellum, polymerization reactions, expelled slime from nanopores, helical motion and shape changing. This section discusses new materials, fuels, geometries, surfaces, fabrication methods and NMs’ efficiencies recently demonstrated for autonomous propulsion of NMs (Figure 3a–j). Biological nanomachines are able to utilize extremely efficient chemo-mechanical energy coupling conversion mechanisms, providing a high specific power [47]. Moreover, a bacterium uses only 2% of its total energy for swimming [48]. A very large number of metals and enzymes can be used to catalyze reactions and power autonomous movement at the micro- and nanoscale. Depending on the shape of the object, the placement of the catalyst and balance of forces different kinds of movements and trajectories can be achieved. Several motive mechanisms have been previously reported: self-diffusiophoresis, self-electrophoresis, bubble recoil and surface/interfacial tension, which were previously not observed in biology.

### 2.1. Improvement of Nano-/Micromotors’ Materials, Geometry, Surface and Motion

It is clear that previous problems of propulsion at a very low Reynolds number have been solved with micromotors, which now move faster than bacteria. Rapid progress in exploring new materials and improved geometries, surfaces and motion of nano-/micro-motors, engines, rotors and pumps began in the pioneering nanomotors research groups of Paxton, Sen, Mallouk and co-workers [2], Ozin and co-workers [3] (Figure 3a). Zhao and co-workers demonstrated applications of graphene oxide (GO/Ti/Pt) microjets by direct evaporation of metals on graphene surface, which self-assembled into tubes due to a weak bonding between GO layers [49]. In a similar study, Bandyopadhyay and co-workers presented glass beads as GO coated spherical motors [50]. Wang’s group demonstrated electropolymerized outer layers, such as polypyrrole (PPy), poly (3,4-ethylenedioxythiophene) (PEDOT), and polyaniline (PANI) on various catalytic surfaces (Ag, Pt, Au, Ni-Pt alloy) [51]. Fischer’s group experimentally proved self-propulsion of the smallest Janus particles with diameter as small as 50 nm (Figure 3c), which, however, were highly influenced by strong Brownian forces [42]. Mei and co-workers applied Atomic Layer Deposition of TiO_2_/Pt Nanotubes to fabricate the smallest nanorockets with diameter around 30 nm (Figure 3i) [37]. Metal-polymer hybrid micromachines with bending and rotational motions based on stacking cationic poly (allylamine hydrochloride) (PAH) and anionic poly (acrylic acid) (PAA) were prepared by layer-by-layer technique [52]. Recently, new geometries and materials were used for catalytic locomotion, including core-shell nanowires, where it was found that self-diffusiophoresis could have a profound influence on the motion [53]. Interestingly, bubble-generation is not observed in smaller motors, where gaseous reaction products can diffuse out of surface quicker than bubble growth/nucleation can occur. Propelled NMs consisting of spherical Au/Ag/Pt nanoshell bubbles were realized by deposition of metals and a subsequent etching of spherical core particle (Figure 3f) [45]. Integration of several nanojets on a larger substrate was demonstrated using layer by layer deposition and under-etching techniques [54]. Theoretical models were developed to describe hydrodynamics of locomotion of both nanowires and engines by imbalance forces and to better understand propulsion mechanisms [55,56,57]. Recently, a low-surface-energy (LSE) layer combined with rough surfaces was fabricated on the outer surface of NMs for an efficient reduction of the fluidic drag force [58]. Au-mesoporous silica NMs powered by a hydrolysis reaction of aqueous NaBH_4_ and KBH_4_ and common H_2_O_2_ fuel [59] are particularly attractive for a micro/nano-carrier system [60]. Platinum-loaded NMs were prepared to power small under-water vehicles by deposition of platinum nanourchins (PNUs) onto cellulose (MFC) films via reduction of chloroplatinic acid (H_2_PtCl_6_) with formic acid (HCOOH) [61]. Metal-organic frameworks (MOF) was applied as motor’s material [62]. It is known that MOF can have an ultrahigh surface area up to several thousand square meters per gram of material (m^2^/g), which is particularly attractive for loading of a high surface area of catalysts or a reduction of consumption of expensive materials. Other impressive examples include tri-metallic microcaps [63], manganese oxide [64], carbon allotrope nanomaterials [65], copper-platinum segmented nanobattery [66] and nanoparticle-mediated motion [67]. Li et al. reported Au–Fe/Ni alloy hybrid nanowire motors, which can achieve speeds up to 850 μm·s^−1^ or 157 BL·s^−1^ [68]. 

### 2.2. Improvements of Fabrication Methods 

Paradigm-shifting fabrication methods represent an important research direction for NMs, which include (i) improvement of fabrication methods and (ii) reduction of fabrication time. Usually, a standard photolithography procedure, followed by e-beam deposition of materials and optional supercritical point drying, was used at the beginning to prepare microjet engines based on strain-engineered rolled-up nanomembranes. However, this procedure takes many hours of fabrication. These methods can be contrasted with well-established fields of microfluidics and self-assembly, where NMs can be generated under ultra-fast fabrication conditions with desired properties and targeted for specific applications. Rapid progress of NMs fabrication has been discussed by Wang and Pumera, including electrochemical/electroless deposition, membrane template-assisted electrodeposition, asymmetric bipolar electrodeposition, physical vapor deposition, glancing angle deposition, self-scrolling method for helical NMs, three-dimensional direct laser writing and layer-by-layer assembly [69,70]. Other impressive fabrication methods include different components in oil-in-water droplets, followed by emulsification, solidification and direct assembly of asymmetric catalytic/magnetic NMs [71]. Examples of other methods are Co-Pt/Au motors with a three-step applied electrochemical potential process [72], shape-controlled fabrication of the polymer-based motors based on the polydimethylsiloxane template [73], template electrosynthesis of graphene microengines [74], layer-by-layer assembly technique in combination with micro-contact printing [75], evaporation-induced self-assembly using controllable crystals of ferrocene-based metal–organic (Fc-Ala-BCB) materials [76], and polymer NMs with doped Pt nanoparticles/carbon nanotubes [77]. 

### 2.3. New Fuels 

Biology employs both “fuel free” Brownian motion and chemically powered motion to support viable functions of organisms. There is much to learn from biological motors, which often operate on very simple and environmentally clean fuels, such as ions and protons. Initially, only hydrogen peroxide was used as a fuel for NMs; however, recent progress in this area has provided a dramatic increase in the choice of a variety of fuels [78]. For instance, soft-oxometalates using dithionite as a fuel, where the redox active Mo^VI^ sites of soft-oxometalates (SOMs) were applied to oxidize dithionite and generate SO_2_ for propulsion [79], magnesium-water reaction [80], motion of graphene swimmers in pure water [81], vapor-driven propulsion [82], hydrogen-bubble driven zinc material in strongly acidic media (Figure 3b) [46], moisture-activated torsional graphene-fiber motor [83], and water-powered cell-mimicking Janus motor [84]. Novel biocompatible fuels were reported using carbonate-based Janus particles propelling in acidic environments [85]. Biofuels are discussed in more detail in the biological section of this review. Not only materials, but effects of ionic screening, Debye lengths, dissociation in solvents [86] and different surfactants are crucial for the motion of NMs [87,88].

### 2.4. Efficiency

Similar to macroscale motors, an efficiency of NMs is highly important for achieving high speeds and long working time [89]. Biological nanomotors are very efficient in chemo-mechanical coupling without the need of an intermediate step for chemical-to-electrical-to-mechanical conversion. Biological nanomotors burn only a few fuel molecules to power mechanical actuation and thus self-propel almost at almost “no cost”. Many other parameters also influence the efficiency of man-made NMs, including micromotors’ geometry, size, materials, temperature, fuel composition and mechanisms of motion.

Valveless microbubble-driven micropumps without moving parts represent a distinct advantage over standard energetically costly pumps [90]. Pumping of fluids can be achieved simply by microbubbles driven by interfacial tension in microtubes. The addition of a surfactant is usually the only requirement for reducing the surface tension and stabilizing the microbubbles. Assuming incompressible, Newtonian and laminar fluid flows through a tube, a pressure drop can be calculated using the Hagen–Poiseuille equation, which states that the flow rate is proportional to the radius of the tube to the fourth power. In this case, a small decrease in the diameter of the tube yields a significant decrease in flow rate

(1) $$\Delta P=8\mu LQ/\pi R^{4}$$

where R is the tube radius, Q is the volumetric flow rate, L is the length of tube, μ is the dynamic viscosity and ΔP is the pressure difference between two ends. Equation (1) is also employed in better understanding of medical delivery and intravenous access of fluids. 

Mallouk and co-workers recently discussed possible efficiency loss mechanisms of NMs with different shapes: wire, tubular and helical structure. So far, the efficiency of NMs was reported on order of 10^−9^ [91]. Solovev et al. found a factor of ×1000 enhancement of microbubble nucleation and growth in tubular microcavity/pumps [92]. When fixed on the surface, microtubes with diameter 5–10 µm and length 30–1000 µm function as catalytic micropumps by decomposition of hydrogen peroxide into oxygen microbubbles and water. Micropump efficiency depends on the minimum fuel concentration for nucleation and stable generation of oxygen microbubbles in catalytic micropumps according to the reaction: 2H_2_O_2_ → H_2_O + 2O_2_. Catalytic microtubes played the role of tubular microcavity for gas collection, bubble nucleation, fluid pumping and ejection of oxygen microbubbles during the decomposition of hydrogen peroxide fuel into oxygen and water. This finding helped to reduce concentration of hydrogen peroxide fuel for microbubble-induced pumping of fluid to 0.009 vol % [92]. In comparison to shorter microtubes (diameter 5–10 µm, length 20–30 µm) by using longer microtubes (diameter 5–10 µm, length 100–1000 µm), it is possible to reduce the threshold hydrogen peroxide concentration for generation of microbubbles. Gao et al. reported efficient microengines fabricated using template electrosynthesis of polyaniline/platinum microtubes [93]. Microengines were 1–2 μm in diameter and 8 μm long self-propelled at 350 BL·s^−1^ in 0.2% of H_2_O_2_. This finding supports the statement that the aspect ratio of tubular micro-cavity is highly important for bubbles generation. 

To better understand efficiency of microbubble-driven catalytic micropumps, we begin by simply estimating the time required for molecular diffusion over realistic distances intrinsic to micropumps. Catalytic micropumps operate using a chemical microreactor, where molecules react and reach opposite walls of the pump much faster than in larger pumps (mm–cm*-* scale). Typical mixing time in a micropump is a second or less, where gas/liquid can be supersaturated in a short period of time. By diffusion time, we can calculate how fast molecules can cross the diameter of pump according to the following equation, $t_{mix}=D^{2}/d$, where *D*—is the diameter of the pump, *d*—is the diffusion coefficient (for example, if *d* = 2.1 × 10^−9^ m^2^/s for O_2_ in water and *D* = 10 µm, *t* = 50 ms). 

Other ideas to increase efficiency consist of new chemical fuels and higher catalyst turnover rates. For instance, Gao et al. experimentally proved that iridium-based Janus NMs could self-propel in ultralow levels of fuels [94]. Esplandiu and co-workers discussed fascinating new control parameters of electrochemical motors and pumps, where the surface potential of self-electrophoretic motors/pumps does not set only the electrochemical double layer, but the strength/direction of proton diffusion flux from the anode to cathode [95]. Increasing surface area is another effective strategy to reduce loading of expensive catalysts and increase efficiency of motors. Pumera’s group used a high surface area iridium-based graphene motors at a low catalyst loading (0.54 at%) [96]. Mei’s group demonstrated efficiency increase using a higher surface area by nanoparticle-decorated tubular microengines using atomic layer deposition, leading to ultrafast speeds up to 3200 μm·s^−1^ [97]. Mei’s group designed a nanoporous reactor/microengine using nanoporous template to improve accessibility of reactants through the reactor walls and larger surface area [98]. Manjare et al. found that the hydrophobic surface of NMs is important to accelerate Janus particles due to possible depletion of water layer and interaction between generated reaction product (oxygen) and hydrophobic surface, leading to slip boundary condition and enhanced reaction rates [99].

Efficiency of electrophoretic nanowire-based nanomotors can be also increased. It is known that pumping of fluids in micro-/nanochannels is usually done not by using external pressure-driven flow, but by electrokinetic flows. For example, Rogers, Adams and Pennathur calculated difference in pressure driven flow necessary to drive water in a nanotube (100 nm in diameter) versus a macrotube (1 m in diameter) at the same flow speed of 1 m·s^−1^ [24]. The reported difference in pressure per length was 14 orders of magnitude higher for a nanotube (2 × 10^12^ Pa/m for nanotube, 0.02 Pa/m macrotube). On the other hand, a very small electric field is needed to induce the flow of fluid through the nanotube (12 fA current for 100 nm diameter tube, accepting the double layer ~3 nm, the zeta potential *ζ* = −100 mV, the relative permittivity of water *ε_r_* = 78.3) [24]. This can explain why an increase of conductivity of anode/cathode lead to high speeds of propulsion of bimetallic nanowire-based nanomotors due to efficient pumping of fluids induced by self-electrophoresis.

### 2.5. New Types of Motion and Trajectories

One very important research direction that needs further exploration is discovering and devising new types of motion by a comprehensive understanding of existing periodic/predictable trajectories and searching for aperiodic/unexpected dynamics for construction of more complicated micromachines. It is known that most NMs move in deterministic straight, helical and rotary trajectories [100,101]. Autonomous trajectories of micromotors can be controlled by the balance of motive–drag forces, shapes and geometrical asymmetry [102,103], effect of catalyst distribution [104], gravitaxis and separation phenomena for mass-anisotropic self-propelling colloids [105]. Interesting observations of periodic oscillatory motion driven by decomposition of H_2_O_2_ using catalase was reported [106]. Several groups observed that catalytic nanorods could spontaneously turn and tumble, which is similar to swimming of bacteria. Schmidt and co-workers showed unidirectional-overloaded transitions in microjet engines [107]. These observations and systematic designs can be the first steps towards efficient designs of more complex autonomous motion.

### 2.6. New Applications

As previously mentioned, NMs can be useful for biological and fluidic applications. Since NMs are coupled to their environment, their motion is influenced by Brownian diffusion, fuels and other processes. Widely used active transport mechanisms can be derived from biological systems albeit with some thoughtful consideration. For instance, Rogers, Adams and Pennathur proposed the idea that if a neuron cell is a meter long it can take thousands of years to deliver proteins from one end of neuron to the other [24]. Control of stepwise motion is another interesting prospect worthy of consideration. Molecules that move step by step while undergoing random collisions can be characterized by a mean free path. During the last few years, a possibility to construct nanoconfined Angstrom-size motors was considered, leading to unprecedented ways of understanding and controlling single molecules and ions [108,109]. It is perhaps most exciting to use NMs for multiple environmental cleaning tasks, where other types of human technology are inaccessible [110]. NMs were recently demonstrated for environmental remediation, pollutant removal and water cleaning application [111,112,113,114,115,116]. Since MOF motors often have a very high surface area, these new roles represent exciting prospects for water purification as shown by Wang et al. (Figure 3d) [43]. Moreover, charged molecules can be adsorbed, transported and separated by polymer motors [116,117]. Furthermore, it is known that micro-/nanobubbles can also be used for the degradation of organic molecules [118]. In this case, kinesin molecular motors can be used to transport proteins in about a week. Other applications include the use of assisted NMs to repair cracks [119], optical nanoscopy [120], pH sensing in motion [121], chemical sensing by quantum dots [122], threat detection [123] and gas sensing applications [124]. On the downside, another question that needs to be addressed is whether micro-/nanomotors themselves represent a new type of contamination or an undesirable byproduct after cleaning a river or lake, for example. 

## 3. Motion Control and Externally Powered Micromotors

External control of autonomous nano-/micro-motors and engines (i.e., sphere, rod, tube and other shapes) can help to achieve fascinating applications such as delivery of micro-cargo, micromachine-enabled assembly of objects in desired configuration and on-chip integration. Followed by our discussion of advanced materials developed for NMs in the Section 2, here we highlight basic methods of NMs’ external control. The first idea includes integration of an additional magnetic layer into NMs. For example, tubular microengines made of rolled-up nanomembranes can contain an additional ferromagnetic layer, leading to a controllable straight, helical, rotary movements of microengines. The second idea considers control of interface between the particle and fluid/fuel. For example, electrochemical modulation of NMs immersed in electrochemical cells can change speeds of NMs due to modification of oxidation and reduction processes, which are taking place on the anode and cathode of bimetallic nanorods. Similar, versatile wireless methods of motion control using light as a fuel source can be integrated, where semiconductor bandgap engineering, semiconductor-metal junctions, better control of electrons, holes, protons, electrons and reaction products are of paramount importance. External light control of motion enables new applications such as cleaning of water in rivers and lakes. In this case, NMs’ knowledge can be often adopted from the well-known industrial methods related to research and discovery of new chemically relevant catalytic materials, processes and applications. Another exciting prospect of NMs is their small size that is particularly attractive for biomedical applications. Externally-powered and stimuli-responsive NMs have been shown, such as NMs driven by ultrasound waves. Multiple achievements of NMs’ control are discussed below in more detail, including magnetic field, light, acoustic field and alternating current.

Dynamic nano-/microstructures, which can be controlled using external fields, already have real applications in separating and biosensing of molecules. For instance, “dynabeads” became a revolutionary technology for liquid separation of bio-materials using superparamagnetic polymer particles with bio-active surfaces to couple various cells and molecules. Therapeutic protocols and diagnostic assays include immunoprecipitation, cell isolation, cell activation and expansion, nucleic acid isolation, mRNA isolation, protein isolation and peptide purification, Streptavidin-coupling and in-vitro diagnostics (IVD) assay development [130]. Another example is biosensing, oscillating DNA helix bonded on surface of electrode enable measurements of affinity and kinetics of DNA-binding proteins, which is not possible to analyze by any other method [131]. Future and important applications of externally controlled NMs can be a “nano-micro-factories”, where NMs can assemble building blocks from the bottom-up. Another envisioned application is chemotactic delivery of drugs.

### 3.1. Magnetically Powered/Controlled Motion

**Figure 4:**
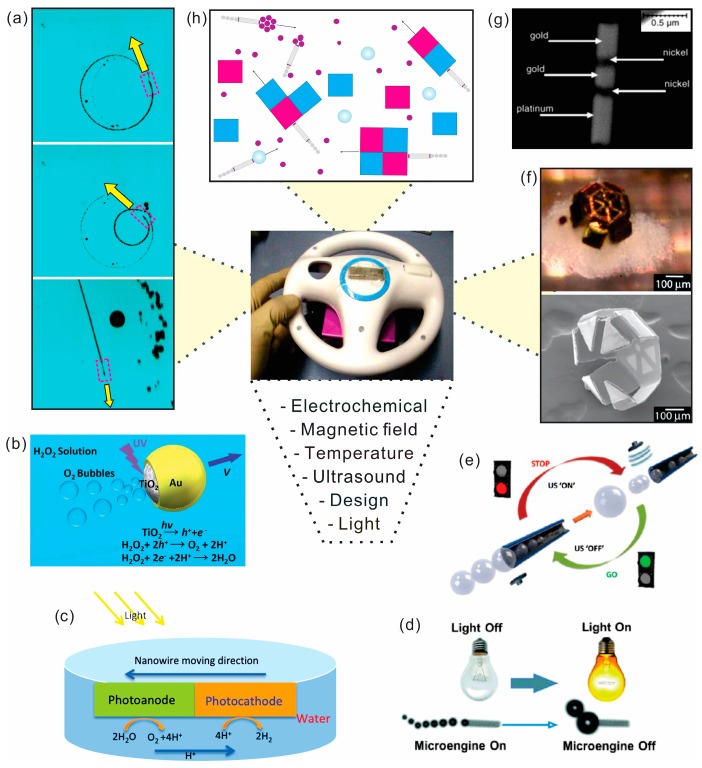
Externally powered “fuel-free” NMs and externally controlled motion of NMs driven by chemical fuels. (**a**) optical microscopy sequences of magnetic control of individual tubular Ti/Fe/Pt microjet in circular and straight motion; (**b**) Janus TiO_2_/Au NM controlled by light. Reproduced with permission from [125], copyright (2016) Royal Society of Chemistry. (**c**) NM driven by photo-electrochemical reaction. Reproduced from [126]; (**d**) microengine switched “on” and “off” by white light by local degradation of hydrogen peroxide fuel above Pt-patterned silicon surface. Reproduced with permission from [127], copyright (2011) Jon Wiley and Sons; (**e**) ultrasound driven NMs. Reproduced with permission from [128], copyright (2014) American Chemical Society; (**f**) magnetic stimuli-responsive microgrippers, design for sampling, analysis of tissue, biomedical minimally-invasive surgery and related operations. Reproduced from [22]; (**g**) the first example of bimetallic nanorods with integrated magnetic segments for external magnetic control of nanomotors. Reproduced with permission from [129], copyright (2005) Jon Wiley and Sons; (**h**) envisioning of a “microfactory”, where externally controlled NMs are used for delivery and assembly of objects. This concept can be realized at the microscale using tubular microjet engines or similar micromotors with high motive power.

Artificial nano-/micro-motors can include an additional segment or layer for (i) external control of catalytic NMs or (ii) full power of NMs using magnetic, electric, acoustic fields, light source and related phoretic, osmotic, electrical, chemical and heat gradients [132,133,134,135,136]. External control helps to perform a task or series of tasks, such as transport, delivery and assembly of micro-cargo payloads and biosensing in motion (Figure 4a,f,g). Groups of Sen and Mallouk were among the first who demonstrated external magnetic control of catalytic nanorods with magnetic segments [129]. Later, Solovev et al. found that an incorporated ferromagnetic layer in rolled-up microtubes can be used to control the motion of microjet engines with magnetic fields [137]. Subsequently, magnetic control of microjets was improved [138,139,140] as well as other NMs including nanowire motors [141], Janus particles [142,143], paperbots [144], liquibots [145], freestyle nanoswimmer [146], fish-like nanoswimmers [147], liquid metal motors [148], flexible and linked superparamagnetic colloidal chains [149], chemo-magnetic structures [150], magneto-electric structures [151], magneto-acoustic structures [152] and chiral nanomagnets [153]. Fuel free NMs have advantages in biomedical applications, since there are no reaction products produced during the navigation [154]. Externally powered NMs are particularly attractive for delivery and assembly of microcargo payloads [155,156], as well as integration in lab-on-a-Chip devices [157,158]. Similar in scope to chemically-functionalized dynabeads, magnetic NMs can be used for cleaning operations and pollutant degradation [159]. Figure 4f shows self-assembling micro-grippers, which can be used to perform engineering functions on the micrometer scale, such as sampling, analysis of tissue, biomedical minimally-invasive surgery and related operations.

### 3.2. Light Powered Motion

Using light source as a fuel to power and control the motion of NMs is a very attractive due to the unique and already existing interactions light has with nano-/micromaterials [160]. However, many challenges and considerations remain such as how to choose and optimize elemental composition of nanomaterials, maximize light harvesting, optimize generation, separate and transport of electrons, holes and protons, match valence band, conduction band, energy band gap, Fermi level of photo-active cathode and anode (if two semiconductors are used), and match nanomaterial-molecule oxidation–reduction potentials, optimize temperature, light intensity and wavelength. Band gap engineering is needed to optimize the performance of light-powered NMs. Light absorption must be maximized while providing sufficient energy to facilitate reduction and oxidation. The thermodynamic potentials for molecules reduction products should fall within the band gap of the metal oxide in order for photo-reduction to occur (Figure 4b–d). Figure 4c shows a single nanomotor powered by photo-electrochemical reactions. Liu discussed an example of junctioned photochemical solar cells, where n-typed hetero-junctioned photoanode (TiO_2_) and photocathode (Pt) can be used [126]. Figure 4d demonstrated light-control over the propulsion of microbubble-driven Ti/Cr/Pt catalytic microengines, where hydrogen peroxide fuel is degraded under local illumination of Si/Pt substrate [127]. Light powered/controlled motion can be related to several categories by using (i) semiconductor photocatalytic [161,162,163,164,165,166,167]; (ii) metallic plasmonic [168,169]; (iii) phototactic [170,171]; (iv) hybrid semiconductor-metallic swimmers [172,173,174,175,176,177]; (v) thermophoresis [178,179,180] and (vi) thermocapillary effects [181]. Other light controlled motors include: dual-light controlled [182] and a spectrally tunable light-driven silicon nanowires [183]. Zheng et al. showed a photo-electrochemically driven nanotree microswimmers with dyes, which can be coded with a distinct spectral response [184], structured light-enabled photoresponsive microstructures [185], chiral colloidal molecules [186], photochemically induced motion of liquid metal marbles [187], self-electrophoretic bimetallic nanomotors and micropumps in halogen media [188]. One of the most interesting applications includes light-driven micro- and nanomotors for environmental remediation of polluted waters [189,190,191] and transport of cargo [192]. 

### 3.3. Acoustically Powered Motion 

Motion powered by acoustic waves became very popular because chemical fuels are not required and, thus, the system is fully biocompatible. Recent reports include artificial acoustically activated flagella [193], acousto-magnetic swimmers [194], metal nanoparticles for acoustic manipulation [195], nanorods trapped in an acoustic field [196], acoustic bubbles for microengines [197,198], acoustic microcannons [199], and nanoshells [200]. Figure 4e shows that ultrasound stimuli can be used to control the movement of bubble-propelled chemically powered PEDOT/Ni/Pt microengines. Applications include intracellular siRNA delivery [201], tissue welding [202] and holograms for acoustics [203]. Chemical and acoustic propulsion of bimetallic micromotors, moving up to speeds of 200 µm·s^−1^, can be realized by ultrasonic standing waves at MHz frequency [204,205]. 

### 3.4. AC Field Powered Motion

One of the biggest advantages of alternating current (AC) powered motion consists of the possibility to modulate flow of electrons, pumping of fluids and control of speeds of motors/pumps without chemical fuels. Moreover, the action of charge separation using external fields is interesting for catalytic nano-/microparticles, where, for example, a catalytic powder can be suspended in aqueous solution for reaction, without physical connections to the surface of electrode. Several groups showed motion of conventional semiconductor diodes and pumps in external AC fields [206], electrochemical rotors [207], nanowire diodes [208], trajectory influenced by AC electrokinetics [209], characterized motion by particle–electrode impact voltammetry [210], and motion of liquid metal Al–Ga–In motors moving at high speed up to 43 cm·s^−1^ under 20 V voltage [211]. 

### 3.5. Other Types of Motion Control

Other effects were observed, for example, motion can be influenced by thermal modulation [212,213,214], solutal and thermal buoyancy effects [215] and photochromic control of bubble-propelled motors by a spiropyran switch [216]. Huang et al. used grating-structured walls for guiding empennages, which improved linear motion of microengines [217]. Bimetallic motors can be accelerated in channels [218,219] and directed in teardrop-shaped posts [220], illustrating their integration into future Lab-on-a-Chip applications.

## 4. Interactive Micromotors: Swarming, Collective and Adaptive Behaviors

Emergent swarming, collective behaviors and Dynamic Self-Assembly (DySA) have been reported for NMs powered by chemical reactions and external fields [221,222]. Similar to biological motor proteins and cells, synthetic catalytic NMs operate by reaction, diffusion and motive forces that can move collectively. Communication by signals, spatiotemporal assembly, chemotactic response, motion either towards or away from chemical gradients, development of patterns and shapes are widely used in biological systems [223]. It is of fundamental importance to have a better understanding of dynamic forces and interactions in natural systems, which can be important for designing more complex NMs [224,225,226]. Short and long range static forces are influenced by (i) the direct motive power of NMs and (ii) released, i.e., “secreted”, ions and molecules in solution, which can establish a long-term “communication” and change behavior of passive and active particles located nearby. Static forces and interactions between atoms, molecules, and micro-/nanoparticles are already relatively well understood (e.g., ionic, electrostatic, covalent, hydrophobic, Van der Waals, magnetic, capillary) [227]. It can explain, for example, why larger and “stickier” molecules have higher boiling points due to larger fluctuating dipoles in molecules. The motive power of synthetic NMs includes transport driven by (1) externally powered/controlled NMs; and (2) autonomous systems driven by diffusion and/or induced forces: self-electrophoresis, self-diffusiophoresis, gas recoil and surface tension driven.

### 4.1. Dynamic Self-Assembly Induced by External Fields and Chemical Reactions

**Figure 5:**
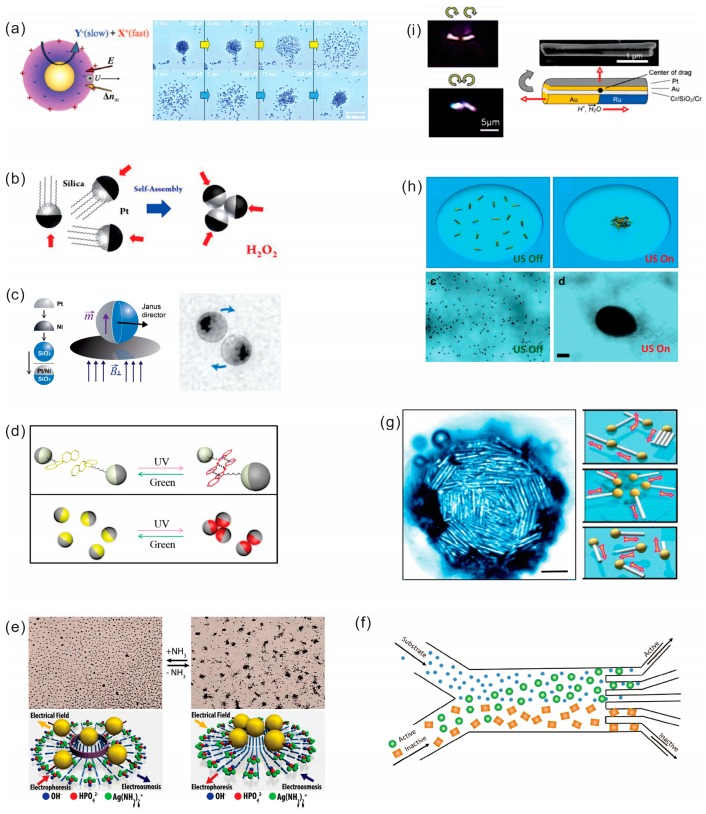
Dynamic self-assembly system based on externally triggered/controlled and autonomous micro-/nanomotors. (**a**) TiO_2_ light driven reversible “microfireworks”. Reproduced with permission from [231], copyright (2010) Jon Wiley and Sons; (**b**) assembly of Janus motors with hydrophobic hemispheres. Reproduced with permission from [235], copyright (2013) American Chemical Society; (**c**) engineered contactless particle-particle interactions. Reproduced with permission from [236], copyright (2017) Jon Wiley and Sons; (**d**) light-induced assembly of spiropyran decorated SiO_2_−Pt Janus particles. Reproduced with permission from [234], copyright (2015) American Chemical Society; (**e**) collective behaviors and response to different stimuli. Reproduced with permission from [247], copyright (2013) American Chemical Society; (**f**) a non-biological chemotaxis phenomena observed for catalytic enzymes in microfluidic channel. Reproduced with permission from [253], copyright (2014) American Chemical Society; (**g**) assembly and interactions between catalytic microtubes in chemical fuels of hydrogen peroxide. Reproduced with permission from [254], copyright (2013) Royal Society of Chemistry; (**h**) reversible swarms self-assembled under acoustic field. Reproduced with permission from [242], copyright (2015) American Chemical Society; (**i**) dynamic interactions between fast chemically-powered nanorotors. Reproduced with permission from [255], copyright (2009) American Chemical Society.

External fields can be used to induce assembly of NMs, such as magnetic, acoustic, alternating electric fields, light (Figure 5a–i). At the same time, NMs can be passive or powered by chemical fuels. Guan’s group reported light switchable colloidal TiO_2_/Pt particles by modified electrostatic interactions [228,229]. Similar, light-induced clustering behavior has been reported by Singh et al. using SiO_2_/TiO_2_ [230], Hong et al. using TiO_2_ [231], and Duan et al. using AgCl particles [232]. The TiO_2_ NMs with a wide bandgap is effective material for water splitting using ultraviolet (UV) photons. Further studies performed by Zhou et al. demonstrated modification effect of Zeta potential due to pH and hydroxyl groups (OH), which influences aggregation of TiO_2_ submicron particles [233]. Upon UV irradiation thermal energy of NMs is reduced from 118.2 kT to 33.6 kT for rutile and from 333.5 kT to 46.1 for anatase, respectively. Spiropyran functionalized SiO_2_-Pt Janus particles in hydrogen peroxide and *N*,*N*-dimethylformamide (DMF) fuel mixture were demonstrated by Zhang et al. [234]. An assembly and transition into multiple motors was observed by electrostatic attraction and π–π stacking between molecules is induced by UV light irradiation (λ = 365 nm), disassembly in monomotors is realized by green light (λ = 520 nm). Wang’s group showed Janus NMs with hydrophobic hemispheres [235]. Gibbs’ group demonstrated contactless long range attractive, short range repulsive and mutual aligned interactions between swimming microparticles [236]. Dynamics of two interacting active Janus particles influence of hydrodynamic interaction due to the propagation of the ionic concentrations and flows influenced by electric force are also reviewed by Bayati’s group demonstrating this principle [237]. Au-Pt catalytic pumps showed repulsion and attraction of silica colloids by the local change of the proton concentration and modification the colloid zeta potential and the electric force [238]. Other external fields used to induce assembly of NMs include Marangoni flow [239] magnetic [240,241], and acoustic fields [242].

For synthetic particles, “quorum sensing” micro- and nanoparticles can release ions, which can build an electric field around the particles due to different diffusion coefficients of anions and cations. Collective swarming behaviors and non-biological chemotaxis of catalytic nano-/microparticles and molecules are interesting fundamental phenomena. Groups of Sen and Mallouk showed biomimetic behavior of particles, such as predator–prey relationship, non-biological chemo- and photo-taxis [243,244]. It is known that biological chemotaxis depends on a temporal rather than on a spatial mechanism. For instance, immotile bacterial cells are too small to measure the difference in the chemical gradient. Bacteria solved this problem by swimming in short runs and sensing of changes in concentrations of attractants or repellents in time rather than in space [245]. When cells detect concentration gradient, they swim in that direction. Similar, synthetic nanomotors can elongate trajectories of their motion in higher concentrations of fuels and align their motion, according to ionic and molecular gradients. It is also known that, in nature, for instance, in stigmergy, intelligent structures can emerge without the direct awareness, communication, intelligence and memory, but due to traces left in the environment that stimulate next actions.

Altemose et al. reported spatiotemporal oscillatory behavior of silver orthophosphate particles under UV illumination in hydrogen peroxide fuel, where an electrostatic self-diffusiophoretic mechanism was connected to alternating electric fields by the reduction and oxidation of silver [246]. It was also shown that NMs with different stimuli might be employed as logic gates. *NOR* Gate with UV and addition of chemical fuel as inputs and collective behaviors as outputs: schooling and exclusion behaviors as *1* and *0*, respectively. From universal *NOR*, other logical gates can be constructed and their combinations, in principle, can be implemented in any digital component [247,248]. Groups of Krishna and Sen recently discovered that active biomolecules, such as enzymes, can increase their diffusion when located in chemical fuels. Followed by Michaelis–Menten kinetics, during substrate turnover, the increased reaction rate leads to the enhanced diffusion of enzymes [249,250]. However, no single conclusion exists about the molecular mechanism observed for enzymes. Groups of Grzybowski and Granick considered networking chemical systems [251] and active colloids with collective mobility [252], suggesting that dynamic interactions can also depend on time, history and feedback control in spatio-temporal scales.

### 4.2. Dynamic Self-Assembly and Adaptive Systems

Dynamic self-assembly systems can be designed based on competing interactions between NMs. In this case, it is possible to estimate characteristic length scales at which collective and swarming interactions can occur. It was observed that catalytic microjet engines at the air–liquid interface of mixed fuels (hydrogen peroxide, propylene carbonate and water) can self-assemble by long lateral capillary forces [256]. Similar behavior was found with biological water striders, which self-organize in colonies and employ water menisci/capillary force to land on solid edges. If motive forces of microjets compete against lateral capillary forces $F_{motive}=F_{capillary}$, one can estimate a characteristic length at which swarming or collective behavior can occur [256]. Lateral capillary force can be simplified to the following equation $F_{c}\sim \gamma R^{2}/L$, where γ is the surface tension, R is the radius of generated bubbles, which shape meniscus and L is the center-to-center distance between particles. Microjet motive force can be described using Stokes law based on recoiling bubbles, $F_{m}=6\pi \mu R\vartheta$, where μ is the fluid viscosity and ϑ is the speed of recoiling bubble. Characteristic length scale at which swarming behavior occurs can be determined, L=γR/6πμϑ. The capillary force can provide long-range interaction up in mm–cm length scale, depending on parameters. It was observed that swarms of microengines decay when concentration of fuel leads to a decrease of bubble radius, thus decreasing the capillary attraction between the bubble-propelled microengines [256]. Similarly, Grzybowski and co-workers described the characteristic distance between the UV-light electrified spheres at the air–liquid interface [257]. In this case, particles attract each other by capillary forces and repel each other by the repulsive electrostatic forces, $F_{q}\sim kQ^{2}/L^{2}$, where k is the Coulomb constant and Q is the charge of each particle. A balance with capillary force leads to the following characteristic length, $L=kQ^{2}/R^{2}\gamma$, when the system can self-organize.

**Figure 6:**
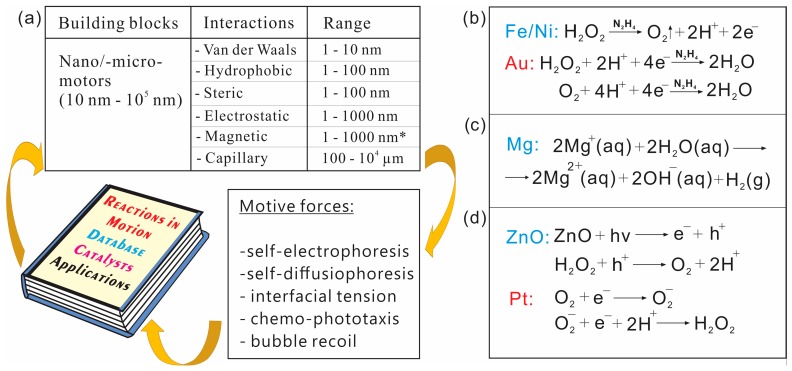
Static and dynamic interactions acting among synthetic nano-/micromotors. (**a**) well-known static interactions between particles (e.g., Van der Waals, hydrophobic, electrostatic, magnetic capillary), which depend on distances between particles, interaction type (* interaction range can vary by changing the size of particles) [251]. Interactions can be perturbed by adding motive force (self-elecrophoresis, self-diffusiophoresis, bubble recoil) to NMs. Databases of known reactions are particularly helpful when working with NMs; one can see a full list of potentially interesting and technologically relevant reactions, catalysts and applications; (**b**–**d**) examples of multi-electrons, protons and photons reactions, which power FeNi-Au, Mg and ZnO-Pt micro-/nanomotors. Released ions and molecules can lead to particle–particle interactions by chemo-and photo-taxis. Combination of individual NMs’ reactions can lead to higher levels of applications, such as fuel cells.

Prominent research challenges consist in better understanding of coupling between chemistry, mechanics, diffusion and active transport using collective micro-/nanomotors in biological and synthetic systems. In his seminal paper in 1952, Alan Turing theoretically predicted patterning by forming concentration gradients in biological systems, where reaction–diffusion driven substances called morphogens (e.g., proteins, small molecules) are non-uniformly distributed in space [258]. The generic mechanism is local activation and long-range inhibition or the diffusion coefficient of the activator (produces itself) must be much slower than the inhibitor. This process was found of paramount importance in development of shapes, forms and skin patterning of animals. Different patterns can be produced by modelling parameters, such as rate of reaction or diffusion. In other words, diffusion can be used to transport chemical signals and set the length scale of patterns. Howard, Grill and Bois suggested that the same holds true for biomolecular motors, which generate forces and the next Turing step could be the mechanochemical basis for morphogenesis [259]. In active transports, the Peclet number is important for both man-made micro-/nanomotors and biological motor proteins. The Peclet number measures ratio of advective to diffusive transport, Pe=ϑl/D, where l is the travelled distance and D is the coefficient of diffusion. The condition required to overcome diffusion is l≥D/ϑ. Usually, if Peclet number is smaller than 1, the diffusion dominates and, if Peclet number is larger than 1, the advective transport dominates. The transport driven only by diffusion leads to the following displacement, $d=\sqrt{Dt}$. If patterning of biological species is driven by reaction and diffusion, the characteristic scale of patterns can be determined, $\tau_{RD}=\sqrt{D/k}$, where k is the rate constant of degradation. However, in many cases, it is not practical to transport by diffusion over longer distances. Howard et al. indicated that chemical signals based only on RD process are often too slow and biology utilizes two strategies: (i) application of motor proteins capable to overcome diffusion and (ii) movement due to mechanical stress (forces exerted by proteins), which can travel 10^6^ times faster than diffusion, i.e., at the speed of sound (1 m/s) [259]. In the case of an advective-diffusive transport by motor proteins, $\tau_{AD}=D/\vartheta$, where ϑ is the speed of motor protein or advective transport. If an advection-reaction only takes place, $\tau_{AR}=\vartheta /k$, where k is the rate constant of degradation. By taking into account viscosity and friction, $\tau_{VF}=\sqrt{\mu /\epsilon }$, where ϵ is the friction coefficient [259]. Figure 6a shows examples of well-known interactions, ranges and nano-/microparticles. Remarkably, long and short-range interactions are vital for dynamic self-assembly of both biological and synthetic nanomaterials.

Chemical databases are already full of innovative and transformative ideas, catalytic materials, tailored by compounds, elements, reactions and utilizations, which can be coupled to motion and assembly of NMs (Figure 6a). Better understanding of flow of electrons, protons, photons and ions during (photo-) catalytic reactions becomes a vital issue for future research directions. Many potentially useful reactions can be considered for design of “indirect” interactions and assemblies of different NMs’. For example, different nanomotors, if combined together, can achieve a higher level of functionality/utility. For example, Li et al. demonstrated application of reaction of hydrogen peroxide decomposition, where reaction products are water and oxygen, Figure 6b [68]. Gao et al. experimentally showed seawater magnesium driven NMs, where hydrogen gas was produced (Figure 6c) [41]. Wang’s group showed symbiosis of two different NMs’ types, production of oxygen and hydrogen fuels by NMs in different chambers for generation of electromotive force in a conventional fuel cells [260]. Dong et al. demonstrated a versatile control of speeds of micromotors using light source, where UV light is used to generate additional electron-hole pairs in ZnO materials for a subsequent enhanced reaction and increased speed (Figure 6) [167]. We believe that NMs can be potentially used for conversion of global warming CO_2_ molecule into solar fuels and related useful by-products, such as methanol and plastics. For example, the following photo-catalytic reactions have been already demonstrated using WO_3_ photoanode and layered double hydroxide (LDH) photocathode, respectively, in the “reversed” fuel cell configuration: $2H_{2}O\to O_{2}+4H^{+}+4e^{-}$ (WO_3_ part), ${\mathrm{CO}}_{2}+6H^{+}+6e^{-}\to {\mathrm{CH}}_{3}\mathrm{OH}+H_{2}O$ (LDH part), to achieve an overall reaction of methanol production using energy of light ${\mathrm{CO}}_{2}+3H_{2}\to {\mathrm{CH}}_{3}\mathrm{OH}+H_{2}O\;$[261].

## 5. Towards Biomedical and Fluidic Micromachines

In recent years, much work on biomedical applications of micro-/nanomotors with high potential for prototype applications has been accomplished: (i) roving biosensors; (ii) drugs, cells delivery; (iii) isolation of pathogens, cancer cells by chemotactic microbots and (iv) cleaning of clogged arteries using microbots [262,263]. Magnetically actuated micro-/nanotools operate fuel-free, such as stimuli-responsive miniature grippers and [22] assisted fertilization by sperm-carrying externally powered microstructure [264]. For NMs powered by chemical fuels in living organisms, a crucial limitation exists: hydrogen peroxide is a highly cytotoxic fuel found in only very small quantities in the body. For this reason, novel biocompatible fuels are desperately needed. Furthermore, the reduction of threshold concentration of fuels does not solve the problem: ideally, the fuel must be a part of cellular metabolic pathways. There is already a well-established field of the U.S. Food and Drug Administration (FDA) approved biomedical drugs, micro-/nano-drops and capsules, which rely on flowing blood streams, passive diffusion, targeted delivery of drugs, nanoparticles and specific surface chemistry for interactions with cells. Weitz’s group demonstrated that microfluidics can further strengthen fabrication of biomedical NMs, since the technology has enabled generation of microdrops at kHz rate, while screening for thousands of potentially useful chemical reactions and new drugs using very small volumes of reagents [265]. Comprehensive results obtained by microfluidics help to mass-produce lipid bilayers, permeable polymers and even nanoparticle-shelled capsules.

### 5.1. Biocompatible Fuels

A number of papers have recently been published on the topic of the motion of nanomotors both in vivo and ex vivo, which can be achieved by exploiting the energy from enzymatic catalysis [266,267,268,269]. Since enzymes normally function in a wide range of environments, both intra- and extracellular nanomotors can possibly be tailored to suit specific mediums with high biocompatibility. Propulsion of nanomotors has been demonstrated with enzymes such as urease and catalase with substrates urea and H_2_O_2_, respectively [270,271]. However, since the conversion of chemical energy into mechanical work is ubiquitous in the organic molecular world, there is a myriad of possibilities in novel nanobiology-related forward movement. Considering that enzymes have a significant turnover rate, even when low concentrations of substrate are available, it makes H_2_O_2_-powered nanomotors feasible in vivo.

The size, shape and material of NMs are of special importance when the motors need to enter the body. For instance, biocompatible nanotubes can be built from an outer coated PEDOT and an inner coated of Pt/Zn [77], making them biodegradable and non-toxic [272]. Besides nanotubes, hollow mesoporous silica Janus nanomotors pose an alternative to nanomotor-design. With their spherical design, Janus nanomotors have high drug-loading capacity as well as great biocompatibility both in vitro and in vivo due to silica being biodegradable, making them an exciting alternative to nanotubes. While nanotubes differ from less than 10 nm to 30 μm in diameter and even more so in length, which gives them a variety of functions, Janus nanomotors are typically 80–500 nm in diameter, which suits cells targeting.

Biofunctionalization and activity of enzymes operate within highly specific conditions. Activity of enzymes depends on their structure: amino-acid polypeptide chains fold in certain ways to form α-helixes, β-sheets or random coil structures as a consequence of hydrophobic interactions between water and the amino acid’s side-chains. Since an attachment of enzyme to a nano-/microstructure requires linker molecules, the protein’s tertiary or quaternary structure might be disrupted to such a degree that it inhibits catalysis. The orientation of the attached enzyme is also of importance, as it must be ensured that the nanomotor does not block the catalytic site or in other ways impose steric hindrance. This requires spatial awareness of the protein and must be accounted for in synthesis [273]. Note that NMs are not limited to nanotubes and Janus particles. For instance, another interesting approach is based on reactions of polymerization. Inspired by the *listeria monocytogenes* that moves by actin-tail polymerization, Pavlick et al. used Grubb’s catalyst to emulate polymerization-movement [274].

### 5.2. Hybrid Bio-Micromotors for Drug-Delivery 

**Figure 7:**
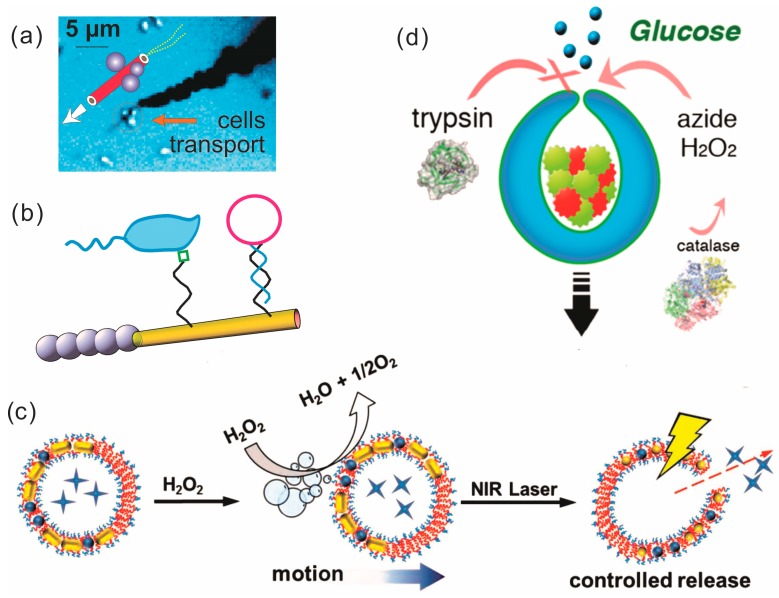
Recent review of bio-hybrid-fluidic NMs for biosensing, drugs delivery and assembly of new micromotors. (**a**) One of the first examples of catalytic nanojet engine transporting Yeast cells in hydrogen peroxide fuel. Reproduced with permission from [292], copyright (2016) American Chemical Society; (**b**) schematic image, biofunctionalized NMs capture and deliver cargo [281]; (**c**) novel architectures consisted of self-assembly of functional nanopraticles in the shell of lipid vesicles. Reproduced with permission from [291], copyright (2015) Jon Wiley and Sons; (**d**) schematic of stimuli-responsive glucose powered soft NM. Reproduced with permission from [263], copyright (2016) American Chemical Society.

Delivery of cells, drugs and proteins by NMs opened new perspectives in application of bio-nanomotors [275,276,277,278,279,280]. Drug delivery in vivo requires target recognition, uptake, movement and eventual release of cargo [281]. Both nanotubes and Janus NMs are implemented in drug-delivery studies. In nanotubes, cargo-uptake can be moderated by the hybridization of adhesive molecules or antibodies to the surface of the tube where molecules can recognize adhesive targets or specific antigens respectively on surface-cell areas, in extracellular matrix or in serum, shown in Figure 7a,b. In Figure 7a, one of the first examples of synthetic catalytic nanoengine assisted transport of yeast cells in hydrogen peroxide fuel is shown. The loading of nanotubes with cargo can also be achieved by pumping a fluid into the mouth of tubes using catalytic reactions [282]. Janus NMs are synthesized through an approach based on microfluidics, and therefore loading of the particles with proteins can be realized during synthesis [283]. Janus NMs can furthermore be filled with nanoparticles e.g., gold nanoaggregates that plays a role in enhancing photothermic tumor therapy [284]. Transport of NMs can be achieved using chemotaxis as previously considered, pH-taxis or external magnetic guidance [285]. Antibody-coatings may also enhance nanomotor-targeting [286]. Realization of cargo delivery is typically done as a consequence of a change in the environment of NMs or using external stimuli. This can be accomplished through chemotaxis or pH-change, where NMs consist of pH-sensitive polymer coatings, which can be dissolved by controllable pH of solution. NMs can also alter the pH locally by using acid as a fuel. Especially in the stomach region, NMs have been considered promising prototypes for drugs delivery and therapeutics [287,288]. Another mechanism of cargo release is through near-infrared (NIR) irradiation, when Au nanoparticles collapse due to photothermic effect and the nanomotors release drugs [289]. This principle is demonstrated in Figure 7c, where Janus particles are assembled with Au and Pt nanoparticles incorporated into the membrane of NMs. Delivery of interior cargo, molecules or particles can be triggered by NIR. A third drug-delivery system exploits endogenous glutathione to break down stomatocyte nanomotors by reduction of disulfide bonds to supply a redox-responsive drug-delivery mechanism [290]. 

Template-free Janus-like vesicles with micromotor function can act as motors for drug delivery; in particular, these NMs can be generated using a microfluidic-based approach. Careful injection of a lipid or block copolymers (BCPs) and inner content, such as inorganic nanoparticles, into a water-based medium through separate channels promotes formation of vesicles: amphiphilic BCP and lipids are self-assembling into vesicle-structures through hydrophobic interactions with surrounding water as it is thermodynamically favorable to minimize the surface-area of amphiphilic substances in a polar solution (Figure 7c). Typical diameters of these vesicles are around 1–2 μm, which depend on amphiphilic materials and the flow rate. For reference, a typical cell has a diameter of 20 μm. Asymmetrical integration of Pt nanoparticles into the membrane can make the vesicles propel forward by H_2_O_2_ decomposition. Encapsulation of intravesicular material can be done co-synthetically through another fluidic channel or post-synthetically by treating the vesicles in the desired intravesicular material [291]. Then, the assembled vesicles can be used for both drug delivery and biosensors.

### 5.3. Hybrid Bio-Motors for Biosensing

Micro- and nanomotors are already employed in the field of biosensors. The majority of biomolecules can be chemically-bonded to the surface of NMs, such as DNA-enabled environmental remediation [293] and small compounds like mercaptohexanol on a gold-surface to form self-assembled monolayers. Hybridization of various bioreceptors to the outer layer of tubular NMs forms a sensing interface on the surface of the motor that can detect biomolecules in situ with short assay time [294]. Information about chemical concentrations can be accessed via motor speed. An example is given from Wang’s group: binding of toxins to the sensory unit of the nano- or micromotor can slow or inhibit catalase activity and thus reduce propulsion of the motor. NMs’ speed is measured as a motion-based signal and chemical concentrations can be accordingly derived [295]. Detection of DNA and RNA can be achieved in a similar fashion. Duplex-formation with a thiolated DNA capture probe results in a binding of the duplex to the nanomotor-surface. When exposed to H_2_O_2_ the Ag-probes are dissolved, releasing Ag^+^-ions in solution that increase the speed of nanotubes in peroxide solution (10% H_2_O_2_) and creates a motion-based DNA/RNA-detection mechanism [296]. In another example, nerve-agents were detected using similar methods [297,298]. Since all of these methods are ex vivo, diagnostics using a lab-on-a-chip devices and micro/nanomotors have great potential to achieve a rapid detection, cheap production and even naked-eye observation. Wang’s group developed such a system, where cortisol, an important stress and clinical biomarker, was detected “on the fly” down to 0.1 μg·mL^−1^ using NMs and horseradish peroxidase [299]. There are still challenges that need to be overcome when applying micro/nanomotors in drug delivery such as poor tissue penetration, effective targeting with little to no off-target effects, and movement against blood flow and full biocompatibility. In biosensing, challenges include specificity and effective assay-application [300]. However, rapid advancements of the nano/microscale motors are expected to have a tremendous impact in the biomedical field.

### 5.4. Towards an Assembly of “Synthetic Cells” Using Microfluidics

**Figure 8:**
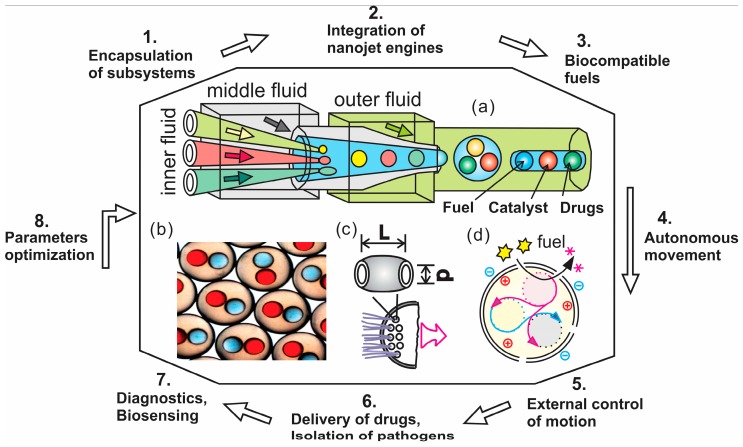
(**a**) A proposed plan to explore fabrication of programmable soft and fluidic NMs using microfluidics. Schematic shows glass multi-capillary device, where inner, middle and outer fluids are used to generate multicomponent drops, capsules for custom-designed NMs; (**b**) example of multicomponent drops, prepared using several syringe pumps. Reproduced with permission from [302], copyright (2012) Royal Society of Chemistry; (**c**) proposal of nanojet engines integration in multifunctional capsules to mimic nanojet powered cells; (**d**) schematic image: towards an assembly of “synthetic cells”, containing coupled fluidic subsystems.

Microfluidics can enable important breakthroughs in fabrication of programmable, evolvable, soft and fluidic micromachines. Since virtually any liquids, biocompatible, soft materials and drugs can be encapsulated in fluidic micromachines, microfluidic techniques hold great potential towards applications in medicine, the environment and on-chip technologies. Future challenges revolve around how to design more complex micromachines and “synthetic cells” from scratch. In 1971, Ganti attempted to characterize the fundamentals of life by conceiving a minimal system able to replicate. According to Ganti, the minimal requirements of a living system are (i) a metabolic chemical network that supplies energy to the collective system; (ii) template polymerization and replication, typically assessed through RNA-templates, and (iii) a lipid membrane enclosing the system. These three subsystems are coupled stoichiometrically, meaning that growth of one part leads to necessary, stoichiometric growth of the other parts though an interlinked, autocatalytic, cyclic system. In the latter case, if a chemical system contains three subsystems, it will be capable of growth and division and, thus, it can fulfill the criteria of life as we know it and observe biological systems [39]. Numerous systems can be designed to meet these criteria, but, in nature, this is observed as classical cell division. Through metabolism pathways, a cell can synthesize nucleotides de novo. High concentrations of nucleotides can contribute to ensuring successful DNA replication. The existence of a duplicate DNA-strand can trigger the expansion of the cell membrane through fatty acid synthesis and ultimately a contractile ring can divide the collective cell-system into two parts [301]. In this scenario, a chemical micromachine can represent a networking metabolic map that can be viewed as thousands of cog-wheels ordered in chemical rather than in geometrical fields. Chemical cycles do not couple with mechanical teeth, but through the fact that the product of one autocatalytic cycle is the “fuel” of another cycle, such as in the case of Belousov–Zhabotinskii reaction. Note that, even though this description is simplified, it demonstrates the principle of the chemoton theory very well. We propose the idea that the chemoton concept can be realized through microfluidics, as input energy can be harvested to synthesize multiple membrane-enclosed systems. Weitz’s group showed that microfluidic devices can be used to prepare reaction vessels for biology, stimuli-responsive capsules, liposomes and polymersomes for diagnostics, drug delivery, design of new materials, isolation of cells and biomolecules. For example, a membrane can be made of lipids or permeable polymers for small molecules to enter and leave the capsule, while keeping larger molecules inside [302]. Figure 8 proposes a research plan, where advanced digital microfluidics can be used to generate multifunctional soft micromachines at a kHz rate.

Rogers, Adams and Pennathur discussed the traffic time, which provides capabilities for very precise and fast mixing of fluids and molecules in micro- drops and capsules [23]. If we estimate the time for two molecules inside the capsule, droplet or cell to meet, i.e., if molecules start off separated by the distance equal to the capsule’s characteristic dimension, *D*

(2) $$t_{traffic}=D^{3}/dr$$

where *d*—sum of diffusion coefficients of both molecules, and *r*—the sum of the molecules radii. For example, a pair of enzymes, 12 and 10 nm in diameter, with diffusion coefficient 10 × 10^−10^ m^2^/s located in 2 µm capsule can meet every 0.4 s. Both *t_mix_* (Section 2.4) and *t_traffic_* are strongly dependent on a capsule characteristic dimension, scaling with *D*^2^ and *D*^3^ [21]—for example, for a capsule with diameter 1 µm *t_traffic_*—is close to a second, for 10 µm—several minutes, for 100 µm—hours. 

## 6. Conclusions 

In summary, we highlighted recent significant results and achievements in the field of NMs, starting from historical overview of simple, complex machines and followed by recent rapid progress made in the field of nano-/micro- motors and engines. Multiple advantages exist for micromachines, which can be further explored and exploited for demanding applications. It includes advantages to overcome Brownian diffusion, outswim high viscosity of fluid at low Reynolds number, and construct better geometries, sizes, fuels for more energy efficient micro-motors and engines. Versatile control of NMs at the sub-microscale using chemical reactions, external fields as well as realization of delivery of objects on-chip and off-chip can be accomplished. Unlike large-scale motors, principles of micro-/nanomotors can go far beyond the classical mechanics analogies. Research progress in better understanding of reaction–diffusion processes, molecular systems, emergent behaviors on the level of individual/collective biomimetic particles and biological enzymes is unprecedented. So far, the only way to learn about NMs was to design, power and observe the motion of these bio-mimetic microparticles, and it remains unknown how many reactions must be tested and what we can really learn from the chemistry in motion. Outlining new horizons in applications, the next decade, in our opinion, is likely to see a considerable rise in application of drop-generating microfluidics to mass-produce fully biocompatible NMs and ultra-sensitive biosensors. A remarkable feature of small size NMs is a high potential for biomedical use, including minimally invasive surgery. In the future, we envision several research directions to advance the field of nano-/nanomachines: (i) better understanding of dynamics of NMs; (ii) understanding of scaling effects: reduction from mesoscale to molecular scale; (iii) application of drop-based microfluidics for fully biocompatible materials; (iv) design of reaction networks for design of more complex programmable and evolvable NMs. For example, if motion of NMs is usually recorded in straight, helical and circular trajectories, it can be related to well-understood periodic or predictable types of motion. Next, it is of high interest to explore transitional behaviors, overloaded and aperiodic motion. Biology is known to operate by nonlinear chemical dynamics, clocks, patterns, oscillations and chaos [303]. Until now, little progress has been given in design of more complex NMs. However, the whole picture of future research directions is still incomplete and NMs’ research methods are often based on exploratory approaches, rather than on systematic designs—our research community has strong evidence that the era of micro-/nanomachinery industry has already started and evolved from its evolutionary stage to the revolutionary phase.
